# Organic Farming Allows Balanced Fungal and Oomycetes Communities

**DOI:** 10.3390/microorganisms11051307

**Published:** 2023-05-17

**Authors:** Bora Nam, Hyo Jung Lee, Young-Joon Choi

**Affiliations:** 1 Department of Biological Science, College of Ocean, Natural Sciences, and Engineering, Kunsan National University, Gunsan 54150, Republic of Korea; kiemoto@gmail.com (B.N.); hjlee@kunsan.ac.kr (H.J.L.); 2 Center for Convergent Agrobioengineering, Kunsan National University, Gunsan 54150, Republic of Korea

**Keywords:** organic farming, fungi, oomycetes

## Abstract

Conventional and organic farming systems affect soils differently, thereby influencing microbial diversity and composition. Organic farming, which relies on natural processes, biodiversity, and cycles adapted to local conditions, is generally known to improve soil texture and alleviate microbial diversity loss compared with that of conventional farming, which uses synthetic inputs such as chemical fertilisers, pesticides, and herbicides. Although they affect the health and productivity of host plants, the community dynamics of fungi and fungi-like oomycetes (under *Chromista*) in organic farmland are poorly understood. The present study aimed to determine the differences in the diversity and composition of fungi and oomycetes inhabiting organic and conventional farm soils using culture-based DNA barcoding and culture-independent environmental DNA (eDNA) metabarcoding. Four tomato farms with different farming practices were selected and investigated: mature pure organic (MPO) via non-pesticide and organic fertiliser, mature integrated organic (MIO) via non-pesticide and chemical fertiliser, mature conventional chemical (MCC) via both pesticide and chemical fertiliser, and young conventional chemical (YCC). Culture-based analysis revealed that different genera were dominant on the four farms: *Linnemannia* in MPO, *Mucor* in MIO, and *Globisporangium* in MCC and YCC. eDNA metabarcoding demonstrated that the fungal richness and diversity on the MPO farm were higher than that on other farms. Both conventional farms exhibited simpler fungal and oomycete network structures with lower phylogenetic diversity. Interestingly, a high richness of oomycetes was shown in YCC; in which, *Globisporangium*, a potential pathogenic group on tomato plants, was abundantly observed. Our findings indicate that organic farming enhances fungal and oomycete diversity, which may provide robust support for maintaining healthy and sustainable agricultural practices. This study contributes to our knowledge on the positive effects of organic farming on crop microbiomes and provides essential information for maintaining biological diversity.

## 1. Introduction

Organic farming systems aim to produce agricultural products by using environmentally sustainable practices. Organic farmers avoid using synthetic pesticides and fertilizers and instead rely on natural and organic inputs, such as compost, manure, cover crops, natural pest control methods, and microorganisms. Organic agriculture is certified by independent organizations in many countries, including the United States and European Union, which set standards for organic production and conduct regular inspections.

Organic farming incorporates crop rotations, cover crops, and organic amendments such as compost or manure to improve soil structure and nutrient availability. Compared with conventional farming, organic practices can enhance soil fertility by increasing soil organic matter and carbon levels but reducing soil erosion [1,2,3]. In addition, it supports higher levels of biodiversity than conventional farming, including increased species richness and an abundance of beneficial insects and soil microorganisms [4,5]. These factors can positively impact soil fertility and biodiversity by promoting sustainable land management practices.

The effects of organic farming on soil microbes are complex and can vary depending on various factors such as farming practices, soil type, and climatic conditions. However, many studies have revealed that organic farming can positively affect soil microbial communities [6,7,8,9,10,11,12,13]. Organic farming often involves the use of natural inputs such as compost, manure, and crop residues that provide a range of nutrients and organic matter to the soil. This promotes the activity and growth of diverse microbial populations in the soil. In addition, organic farming practices, such as reduced tillage, cover cropping, and intercropping, can improve soil structure and increase soil organic matter content, creating a favorable environment for soil microbes. Therefore, organic farming practices lead to higher microbial biomass and activity, as well as a greater diversity of microbial communities, compared to conventional farms [6,14,15], and thus can contribute to a healthy and more sustainable soil ecosystem.

Arbuscular mycorrhizal fungi (AMF) are important soil organisms that form symbiotic associations with the roots of most plant species. They enhance plant growth, improve nutrient uptake, and confer resistance against various stressors. Although the relationship between organic management and AMF diversity is complex, organic management can enhance the diversity and abundance of AMF assemblages in different agricultural contexts, such as in sorghum [16], rice cropping [17], and agroforestry system [18], compared with conventional management practices. However, other studies reported no significant differences in AMF diversity between organic and conventional management systems. For example, AMF diversity did not differ significantly between organic and conventional cropping systems in onion [19] and wheat [20] fields.

Pesticides or herbicides can play a positive role by selectively targeting harmful pathogens and pests that damage crops. The impact of pesticides on soil microorganisms depends on a range of factors, including the type of pesticide used, the dosage and frequency of application, and the specific microbial populations present in the soil. However, many pesticides can have direct and indirect adverse effects on microbial biomass, community composition, diversity, and the function of soil microbial communities, including bacteria, fungi, and archaea, by killing beneficial microbes that play a key role in decomposing organic matter and releasing nutrients that plants need to grow and contribute to soil health and fertility [21,22,23]. Fungicides significantly affect soil oomycetes and fungi, and the total number of oomycetes and fungi is considerably lower in fungicide-treated soils than in untreated soils [24,25,26].

This study aimed to determine the diversity and composition of soil-borne fungi and fungi-like oomycetes inhabiting organic and conventional farming using culture-based DNA barcoding and culture-independent eDNA metabarcoding. Fungi and oomycetes strongly affect host plant health and productivity. However, the community dynamics of fungi and oomycetes in organic farmlands used for major food crops are poorly understood. The present study investigated the diversity and composition of fungal and oomycete communities in organic and conventional tomato farms, which could contribute to our knowledge of crop microbiomes and the effects of organic farming on their structure.

## 2. Materials and Methods

### 2.1. Sampling and Sites Description

Soil samples were collected in March 2022 from tomato (*Lycopersicon esculentum*) farms located in agricultural areas of Gunsan and Iksan in Korea. We collected five soil samples from each of four distinct farming practices, maintaining a 1 m distance between samples: a mature pure organic (MPO) farm using non-pesticide and organic fertilizer, a mature integrated organic (MIO) farm using non-pesticide and chemical fertilizers, a mature conventional chemical (MCC) farm using both pesticide and chemical fertilizer, and a young conventional chemical (YCC) farm. For each farm, the replicate samples were combined, resulting in a total of four pooled samples. The MPO, MIO, and MCC farms have continuously produced tomatoes for over ten years, whereas the YCC farm began to produce crops approximately two years ago. Information on the collection sites, climatic conditions, and soil chemical characteristics is summarized in Table 1. The soil properties were analyzed by the Agriculture Technology Promotion Agency in Korea. Information on the climatic conditions was collected from the weather data portal of the Korean Meteorological Administration (accessed on 1 March 2023 at http://data.kma.go.kr).

### 2.2. Culture-Based DNA-Barcoding

To isolate fungal and oomycetes strains from soil sediment, a simple plating technique at a 1:10 soil dilution was used with potato dextrose agar (PDA; Difco, Detroit, MI, USA) and 5% V8 agar (V8A; 50 mL clarified V8 juice, 10 g CaCO_3_, 15 g agar, 950 mL deionized water) plates. The plating process was performed in two steps with three replicates for each sample. After incubation of the smear plates for 1–2 days at 25 °C in the dark, hyphal tips were isolated from the outgrowing mycelia and transferred onto new plates. Following colony formation 3–5 days later, newly outgrowing mycelium was isolated once more. The colonies of the isolates on the new plates were classified by their cultural and morphologic characteristics such as shape, colour, surface texture and growth rates. One or two representative isolates with distinct phenotypes were then selected for subsequent analysis. To identify the isolates morphologically, their cultural characteristics were investigated after incubation for 5–7 days, and the microscopic structures were observed under a Zeiss Axio Imager A2 microscope (Carl Zeiss, Oberkochen, Germany). To confirm the morphological identification, genomic DNA was extracted using the MagListo 5M Plant Genomic DNA Extraction Kit (Bioneer, Daejeon, Republic of Korea). The internal transcribed spacer (ITS) rDNA regions were amplified by polymerase chain reaction (PCR) using the primer pairs ITS1/ITS4 [27]. Additionally, the cytochrome c oxidase subunit I (*cox*1) mtDNA of oomycete strains was amplified using OomCox1-levup/OomCox1-levlo [28]. The DNA amplicons were sequenced by Macrogen Inc. (Seoul, Republic of Korea) and purified using an AccuPrep PCR Purification Kit (Bioneer). The sequences edited using the DNAStar software package 5.05 (DNAStar, Inc., Madison, WI, USA) were BLASTed to search for sequences homologous to the National Center for Biotechnology Information (NCBI) GenBank database. The sequences obtained in the present study were deposited in the NCBI database under accession no. OQ706980–OQ707024 for ITS and OQ718330–OQ718338 for *cox*1 sequences. Phylogenetic analysis was performed using the ITS rDNA dataset for fungal strains and the *cox*1 mtDNA for oomycete strains. The datasets were created by aligning the sequences of the strains obtained in the present study and previously published authentic isolates in the NCBI GenBank using the G-INS-i algorithm [29] of MAFFT 7 [30]. Maximum likelihood (ML) and minimum evolution (ME) inferences with the Tamura-Nei model were used to construct phylogenetic trees using MEGA X [31]. Bootstrapping (BS) was performed with 1000 replicates.

### 2.3. Culture-Independent eDNA Metabarcoding

Total genomic DNA from each soil sample was extracted from 0.2 g of soil using a FastDNA^®^ Spin Kit (MP Biomedicals, Santa Ana, CA, USA) following manufacturer instructions. The internal transcribed spacer 2 (ITS2) rDNA region of fungi and oomycetes was amplified with the primer pair ITS3 (5′-(A)GCATCGATGAAGAACGCAGC-3′)/ITS4 (5′-(B)TCCTCCGCTTATTGATATGC-3′) [27] and ITS3oo (5′-(A)AGTATGYYTGTATCAGTGTC-3′) [32]/ITS4 [27], respectively. The A and B sequences fused to the 5′ primer ends represent the sequencing adapters: TCGTCGGCAGCGTCAGATGTGTATAAGAGACAG and GTCTCGTGGGCTCGGAGATGTGTATAAGAGACAG, respectively. DNA extraction, PCR, and Illumina sequencing were performed and analyzed by Macrogen, Inc.

The amplicons were sequenced on an Illumina MiSeq Platform (Illumina Inc., San Diego, CA, USA) in a 300 bp paired-end format, and amplicon sequence variants (ASVs) were analyzed by Macrogen Inc. The adapter and primer trimming was carried out using Cutadapt (ver. 3.2) [33]. The trimmed reads were processed using DADA2 (ver. 1.18) [34] for read error correction, merging, and denoising to obtain the ASVs sequences. Chimera removal was performed using the consensus method with the remote Bimera Denovo function in DADA2. The reads were grouped into exact ASVs using DADA2. The BLAST+ (ver. 2.9) was performed for each ASV against the UNITE database (ver. 8.2.) [35] to obtain taxonomic information (query coverage > 85%; identity > 85%). The sequences were rarefied to calculate alpha diversity indices. The rarefaction curve and alpha-diversity characteristics of each soil sample, including Good’s coverage, indices of richness (Chao1), and diversity (Shannon and Gini-Simpson), were calculated using Quantitative Insights into Microbial Ecology (QIIME) (ver. 1.9) [36]. Significant differences in read abundance, ASV richness, and diversity across soil samples were assessed using Kruskal–Wallis rank sum tests with a Dunn Post Hoc test and Bonferroni correction. The community compositions in each soil sample are shown in a heatmap using the heatmap package in R (ver. 4.2.3). The relationships between the samples were analyzed by the hierarchical clustering analysis using the Unweighted Pair Group Method with Arithmetic mean (UPGMA) based on the beta (β) diversity estimated in the Vegan package of R. The linear discriminant analysis (LDA) effective size (LEfSe) algorithm [37] was used to determine significantly different classes in fungi and genera in oomycetes from organic and conventional farms. Data containing the relative abundance of classes or genera were imported into LEfSe (ver. 1.0) on the web-based Galaxy, with logarithmic LDA scores >2.0. Box plots were constructed using the ggplot2 package in R [38] of R. The sequence datasets were deposited in the NCBI Sequence Read Archive (SRA) database under accession no. PRJNA949862.

## 3. Results

### 3.1. Distribution of Cultureable Fungi and Oomycetes

**Figure 1:**
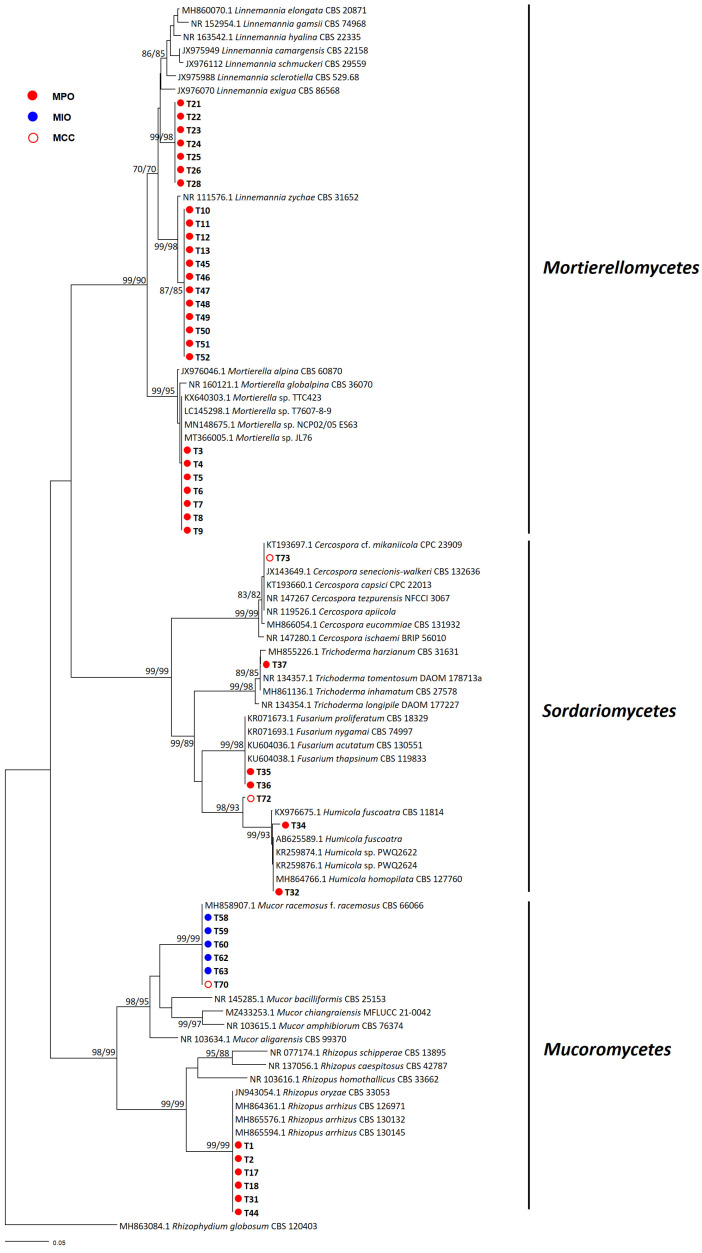
Phylogenetic tree of fungal isolates inferred from the minimum evolution (ME) and maximum likelihood (ML) analyses of the internal transcribed spacer (ITS) rDNA sequences. Bootstrapping values (minimum evolution BS/maximum likelihood BS) higher than 70% were given above or below the branches (1000 replicates). *Rhizophydium globosum* was used as outgroup. The strains isolated in the present study are shown in bold. The scale bar equals the number of nucleotide substitutions per site.

Fungal genera of *Mortierellomycetes*, *Sordariomycetes*, and *Mucoromycetes* were isolated from the MPO farm, namely *Fusarium*, *Humicola*, *Linnemannia*, *Mortierella*, *Rhizopus*, and *Trichoderma*. *Linnemannia* and *Mortierella*, both belonging to *Mortierellomycetes* were exclusively found from the MPO farm. *Mucor*, a member of *Mucoromycetes*, was identified on both the MIO and MCC farms, while *Humicola* was also isolated from the MCC farm. No fungal isolates were found at the YCC farm. The rDNA ITS phylogenetic tree (Figure 1) shows the fungal distribution across the MPO, MIO, and MCC farm. The strains in the present study formed a well-supported group with the reference sequences of authentic isolates obtained from the BLAST search and exhibited a maximum supporting value of 98–99% in both ME and ML analyses.

**Figure 2:**
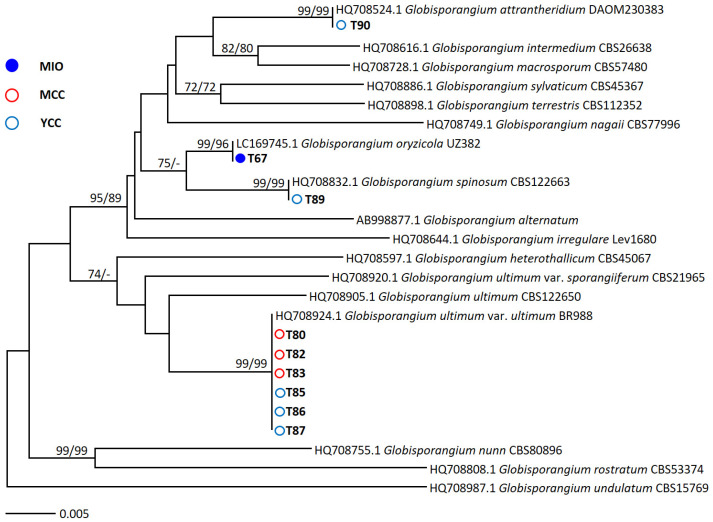
Phylogenetic tree of oomycetes isolates inferred from the minimum evolution (ME) and maximum likelihood (ML) analyses of cytochrome c oxidase subunit I (*cox*1) sequences. Bootstrapping values (minimum evolution BS/maximum likelihood BS) higher than 70% were given above or below the branches (1000 replicates). *Globisporangium undulatum* was used as outgroup. The strains isolated in the present study are shown in bold. The scale bar equals the number of nucleotide substitutions per site.

Oomycetes were found in the MIO, MCC, and YCC farm. The species *Globisporangium oryzicola* was discovered from the MIO farm, whereas *G. attrantheridium*, *G. spinosum*, and *G. ultimum* were isolated from the YCC farm. From the MCC farm, only *G. ultimum* was found. No oomycetes were found in the MPO farm. The *cox*1 tree (Figure 2) presents a phylogenetic relationship between the oomycete isolates obtained from the present study and their reference isolates from the BLASTn search, with high support values of 96–99% in the ME and ML analyses.

### 3.2. Fungal Composition and Diversity

**Figure 3:**
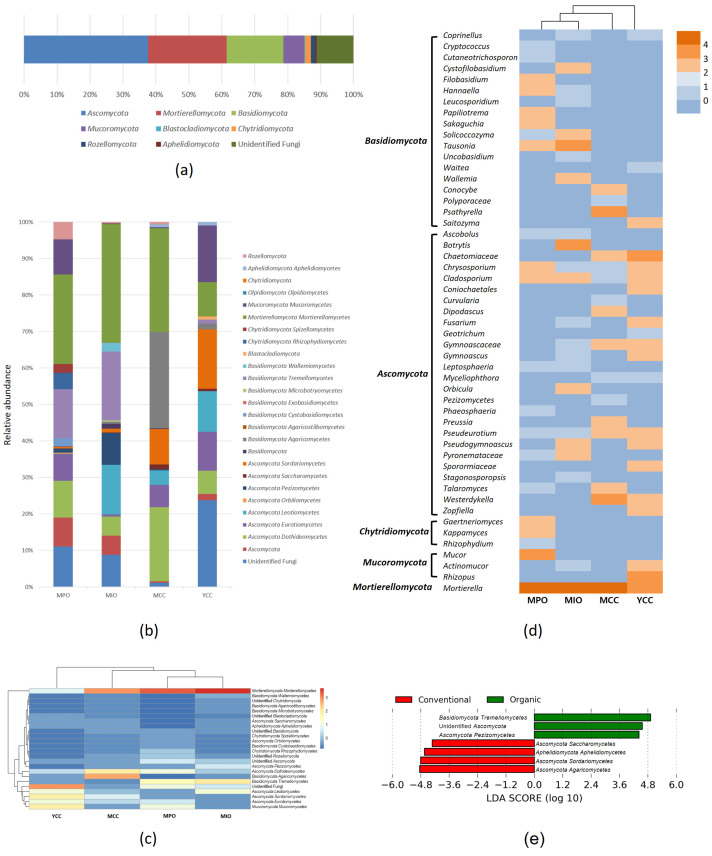
(**a**) Relative abundances of fungal phyla across four tomato farms; (**b**) Relative abundances of fungal classes on each tomato farm practice; (**c**) UPGMA distance sample cluster tree and the heatmap for fungal community composition based on class in each tomato farm practice; (**d**) Relative abundances of top 20 fungal taxa in total fungal communities in each farm; (**e**) Linear discriminant analysis effect size (LEfSe) analysis of fungal classes with differential abundance between organic and conventional farms.

A total of 473,451 raw sequences were obtained from ITS3/ITS4 dataset across the soil samples, and the sequences ranged from 14,480 to 30,595 high-quality sequences per soil sample. The sequences were rarefied to 14,480 reads per sample, and the final result consisted of 101 fungal ASVs across the samples. The dominant fungal phyla across all samples were *Ascomycota* (37.7%), *Mortierellomycota* (23.74%), and *Basidiomycota* (17.2%), followed by unclassified fungi (11.17%), *Mucoromycota* (6.4%), *Chytridiomycota* (1.74%), *Rozellomycota* (1.41%), *Aphelidiomycota* (0.43%), and *Blastocladiomycota* (0.2%) (Figure 3a). Both organic farms, MPO and MIO, were primarily dominated by *Mortierellomycetes* (24.55% in MPO and 32.59% in MIO) and *Tremellomycetes* (13.59% in MPO and 18.85% in MIO). *Mortierellomycetes* and *Tremellomycetes* belong to *Mortierellomycota* and *Basidiomycota*, respectively. The dominant classes on the mature conventional farm MCC were also *Mortierellomycetes* (28.37%) but followed *Agaricomycetes* (26.51%) in *Basidiomycota*. The relative abundance of *Agaricomycetes* was higher in the MCC farm, compared to other farms and was not observed in the MPO farm.

The young conventional farm YCC did not exhibit dominance by *Mortierellomycetes* nor *Agaricomycetes*. Instead, unidentified fungi (23.76%), *Sordariomycetes* in *Ascomycota* (16.23%), and *Mucoromycetes* in *Mucoromycota* (15.55%) were abundant (Figure 3b,c). LEfSe analysis showed significant differences in the relative abundances of *Tremellomycetes*, *Agaricomycetes*, and *Sordariomycetes* between organic (MPO and MIO) and conventional farm (MCC and YCC) (Figure 3e). Several genera of *Tremellomycetes* were found inhabited the MPO farm; *Bullera*, *Cryptococcus*, *Cutaneotrichosporon*, *Filobasidium*, *Hannaella*, *Papiliotrema*, *Solicoccozyma*, *Tausonia*, *Udeniomyces*, and *Vishniacozyma*. The genera *Hannaella*, *Solicoccozyma*, and *Tausonia* were also present in the MIO farm, with *Tausonia* being most abundant in the organic farms. No *Tremellomycetes* was found at the MCC farm. The genus *Saitozyma* was the only member of *Tremellomycetes* in the YCC farm. Contrastively, the relative abundances of *Agaricomycetes* and *Sordariomycetes* were significantly higher in both conventional farms. Most of their member were observed exclusively in the MCC or YCC farm, except for the genera *Fusarium*, *Coprinellus*, and *Uncobasidium* which were also found in the MIO farm. The dominant taxa *Psathyrella* of *Agaricomycetes* and unidentified *Chaetomiaceae* of *Sordariomycetes* were abundant only in the MCC and YCC farm.

In total fungal communities, the dominant genus on both organic farms was *Mortierella* in *Mortierellomycota*. (Figure 3d). In the MPO farm, *Mucor* in *Mucoromycota*, *Kappamyces*, *Gaertneriomyces* in *Chytridiomycota*, and *Sakaguchia*, *Papiliotrema*, *Filobasidium* in *Basidiomycota* were also abundant; however, these genera were not found in other farms. *Botrytis* and *Orbicula* in *Ascomycota*, and *Cystofilobasidium* and *Wallemia* in *Basidiomycota* abundantly inhabited only the MIO farm.

**Figure 4:**
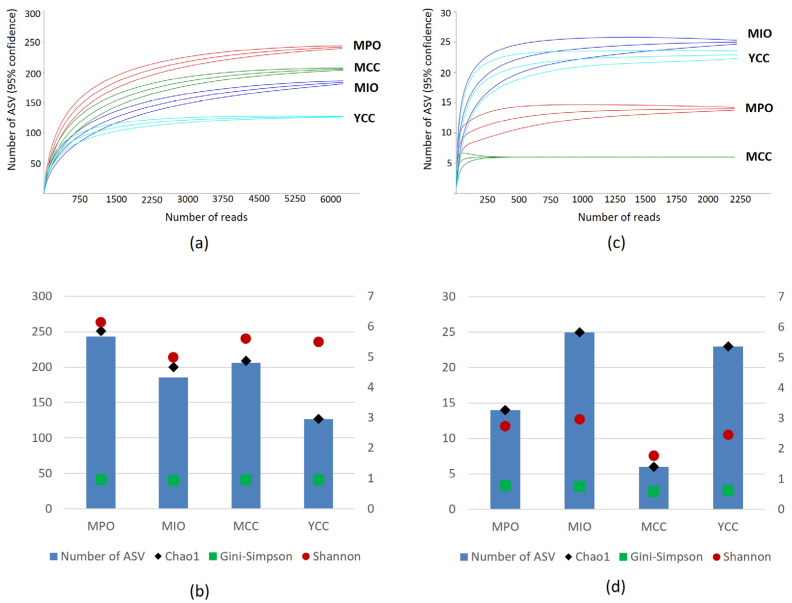
(**a**) Rarefaction curve resulted from the metabarcoding analysis using ITS2 region sequences for Fungi with 95% confidence interval.; (**b**) Level of ASV richness, Chao1, Gini-Simpson, and Shannon index for fungal community in each tomato farm practice; (**c**) Rarefaction curve resulted from the metabarcoding analysis using ITS2 region sequences for oomycetes with 95% confidence interval; (**d**) Level of ASV richness, Chao1, Gini-Simpson, and Shannon index for oomycetes community in each tomato farm practice.

The composition of fungal genera in conventional farms differed from that in the organic farms. *Mortierella* was dominant in the MCC farm, but *Psathyrella* in *Basidiomycota* and *Westerdykella* in *Ascomycota* were also enriched in the MCC farm, whereas these genera were not found in the organic farms. The most abundant genus in the YCC farm was *Rhizopus.* Besides, *Sporormiaceae*, *Zopfiella*, *Coniochaetales*, and *Geotrichum* in *Ascomycota*, and *Saitozyma*, *Waitea*, and *Coprinellus* in *Basidiomycota* inhabited the YCC farm but were not found in the organic farms. The Good’s coverage index was above 0.99 in all soil samples and rarefaction curves for the ITS3/ITS4 dataset of all soil samples reached the asymptote (Figure 4a), indicating the sequencing reads were sufficient for fungal community analysis. However, ASV richness, Chao1, and Shannon indices were higher in the MPO farm than in the other farms (Figure 4b). Significant differences in richness and diversity were observed across soil samples (*p* < 0.05).

### 3.3. Oomycetes Composition and Diversity

**Figure 5:**
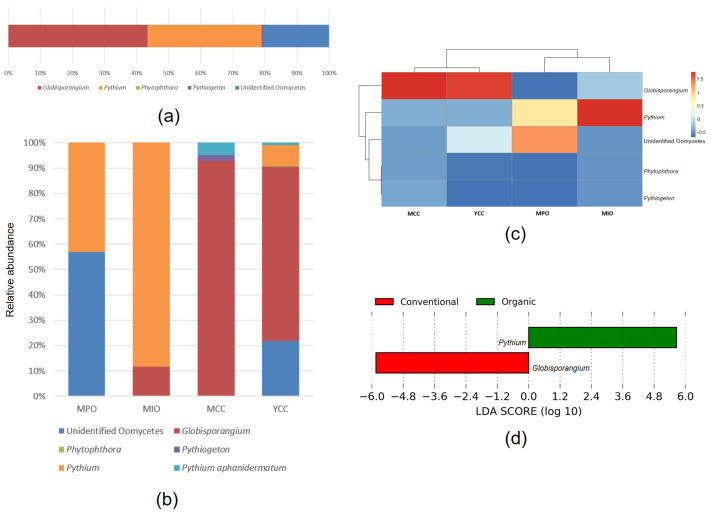
(**a**) Relative abundances of oomycetes genera across four tomato farms; (**b**) Relative abundances of oomycetes on each tomato farm practice; (**c**) UPGMA distance sample cluster tree and the heatmap for oomycetes community composition based on genus in each tomato farm practice; (**d**) Linear discriminant analysis effect size (LEfSe) analysis of oomycetes genera with differential abundance between organic and conventional farms.

High-quality sequences, ranging from 2503 to 16,712 per sample, were obtained from the ITS3oo/ITS4 dataset, yielding a total of 195,050 raw sequences. Sequence data were rarefied to 2503 reads per sample. The final results included 25 oomycete ASVs across all samples. The dominant oomycete genera in all samples were *Globisporangium* (43.34%) and *Pythium* (35.56%), followed by unidentified oomycetes (20.48%), *Pythiogeton* (0.55%), and *Phytophthora* (0.07%) (Figure 5a). However, *Globisporangium* was not observed on the MPO farm, which contained only *Pythium* (42.98%) and unidentified oomycetes (57.02%) (Figure 5b,c). *Pythium* was also predominant on the MIO farm (88.22%). In contrast, the dominant genus in both conventional farms, MCC and YCC, was *Globisporangium* (92.74% in MCC and 68.69% in YCC). Moreover, *Pythiogeton* and *Phytophthora* were found on the MCC (2.28%) and YCC (0.25%) farms, respectively. These genera were not observed on both organic farms.

LEfSe analysis showed that the relative abundance of *Pythium* and *Globisporangium* was significantly different between organic farms (MPO and MIO) and conventional farms (MCC and YCC) (Figure 5d). Good’s coverage indices ranged from 0.99 to 1 for all samples, indicating that the sequencing reads were sufficient for oomycete communities. The rarefaction curves for the ITS3oo/ITS4 dataset of all soil samples reached an asymptote (Figure 4c); however, ASV richness and Chao1 were notably high in the MIO and YCC farms (Figure 4d). The Shannon index was higher in both organic farms than in both conventional farms, and there were significant differences in richness and diversity across the soil samples (*p* < 0.05).

## 4. Discussion

Previous studies have demonstrated that organic practices positively affect soil quality by increasing organic matter and carbon levels compared with conventional farming [1,2,3,4,5] and enhance microbial richness and diversity [6,14,15,39]. The tomato farms investigated in this study have different histories and stand ages, as reflected in their soil profiles. The mineral content, organic matter, and carbon levels in the organic farm were clearly different from those in the conventional farm. Soil from the pure organic farm had the highest level of potassium, phosphorus, calcium, carbon, and organic matter. A culture-independent analysis revealed a more diverse fungal community in the pure organic farm than in the other farms. In addition, various fungal strains were isolated from the pure organic farm using culture-based analysis. Therefore, fungal diversity seems to be related to soil conditions, affected by organic farming practices, although further research is needed to establish the correlation between soil properties and fungal community dynamics.

The present study highlights the differences of fungal community composition between the organic and conventional farms. LEfSe detected a significant differential abundance of fungal groups that were significantly enriched in organic or conventional farm. The organic farms, both pure and integrated, were dominated by *Tremellomycetes*, which are known as jelly fungi. These fungi produce both unicellular yeasts and filamentous fungi during their life cycles and have various lifestyles, such as saprotrophic, fungicolous, and pathogenic. However, they can also encourage host plants to obtain nutrients from the soil [40]. Two dominant genera *Hannaella* and *Tausonia* of *Tremellomycetes* are believed to benefit the host plants and soil environment. *Hannaella* is frequently found on the phyllosphere of diverse plants [41,42], and generates indol acetic acid [41,43], which stimulate plant growth. *Tausonia* produces auxin-like compounds which promotes plant growth but inhibits the growth of pathogenic fungi and oomycetes, such as *Verticillium dahliae* and *Pythium aphanideratum* [44]. Additionally, *Linnemannia*, a genus that may potentially promote plant growth and seed production [45], was isolated exclusively from the pure organic farm through culture-based analysis. These genera were not found in the conventional farm, indicating that the organic farming could potentially lead to a positive shift in fungal composition. The mature and young conventional farms were dominated by *Agaricomycetes* and *Sordariomycetes*. *Agaricomycetes* was significantly more abundant in mature conventional farm, but not observed in pure organic farm. This class contains various species, including ectomycorrhizal symbionts, decomposers, and pathogens [46], but their impact on tomato plants remains unknown. On the other hand, *Sordariomycetes* includes many plant pathogens, among which two *Fusarium* species, *Fusarium oxysporum* and *F. solani*, were found only from the conventional farm and are the most devasting wilt pathogens occurring on tomato that invade the vascular system by colonising the root tissues [47]. Other well-known fungal pathogens of tomato plants, such as *Alternaria*, *Colletotrichum*, *Sclerotium*, *Septoria*, and *Verticillium* [47], were not observed in any of the farms.

Three oomycete genera (*Globisporangium*, *Phytophthora*, and *Pythiogeton*) and *Pythium aphanidermatum* were identified solely in the conventional farm through culture-independent analysis. The genus *Globisporangium* contained clades E–G, I, and J of *Pythium* Pringsheim (nom. cons.) sensu lato (s.l.), which have recently been reclassified as *Elongisporangium*, *Globisporangium*, *Phytopythium* (*Ovatisporangium*), *Pilasporangium*, and *Pythium* sensu stricto (s.s.) [48,49]. Most species of *Globisporangium* are plant pathogens, with *G. ultimum* causing tomato root rot [50]. The genus *Phytophthora* is also associated with tomato diseases, including root rot [51] and late blight [52]. *Pythium aphanidermatum* causes tomato damping-off [53]. Through the culture-based analysis, three plant pathogens *G. attrantheridium*, *G. spinosum*, and *G. ultimum* var.* ultimum* [54,55,56] were mainly isolated from the conventional farm. The increased dominance of *Globisporangium* in the conventional farm may be related to pesticide usage, which negatively impacts on microbial composition. The input of pesticides can hinder the growth and development of soil-borne saprophytes and trigger the emergence of new isolates resistant against these chemicals. This outcome demonstrates that employing pesticides can lead to unfavourable environment for beneficial soil-borne saprophytes, while inadvertently facilitating the proliferation and prevalence of the resistant pathogens.

Interestingly, the pure and integrated organic farms exhibited a dominance of *Pythium* s.s. The abundance of *Pythium* s.s. was significantly higher in the organic farm compared to conventional farm. Primarily, *Pythium* s.s. functions as saprophyte and decomposes organic matter that varies with soil characteristics, especially soil pH, which is an essential factor that affects soil-borne *Pythium* [57,58]. The chemical conditions within organic farm may be interrelated with the prevalence of *Pythium*. In particular, the pH level was substantially lower, and the organic matter was significantly higher in the pure organic farm than in other farms. This could be a contributing factor to the high diversity of *Pythium*.

In line with previous studies [2,3], the present study confirmed that the organic matter and carbon levels are higher in the organic farm compared to conventional farm. Moreover, the fungal ASVs richness and diversity were higher in the organic farm, with the highest levels found in the pure organic farm, corroborating previous findings [6,59,60]. However, no specific association was observed for mycorrhizal fungi or plant growth-promoting fungi [61]. Therefore, low-input farming appears to support a balanced plant microbiome, fostering high biodiversity and soil quality, and thus enabling sustainable agriculture. In contrast, conventional chemical farm using both pesticides and chemical fertilisers displayed a significantly higher relative abundance of plant pathogens compared to the organic farm. As observed in previous studies [9,62], pesticides significantly influenced both saprophytic fungi and oomycetes, leading to a substantial reduction in their diversity in pesticide-treated farms relative to untreated ones. Consequently, these findings indicate that low-input farming helps maintain the plant microbiome balance, contributing healthy and sustainable agricultural systems. However, the current study faced a certain limitation, predominantly due to the small sample size that arose from pooling duplicates, necessitating a more reliance on qualitative comparative analysis. For future studies, it would be beneficial to ensure an adequate sample size for each farming system, as this would enhance the reliability and validity of the present findings. Despite this limitation, this study enhances our understanding of the effects of organic and conventional chemical farming practices on microbiome diversity and offers valuable insights for future research aimed at promoting sustainable agriculture.

## Figures and Tables

**Table 1:** Information on soil collection sites of tomato farms in Gunsan and Iksan in Jeollabuk-do of Korea, climatic conditions, and soil characteristics.

Farming Practices		MPO	MIO	MCC	YCC
**Collection site**	**Stand age**	10 yr	10 yr	10 yr	2 yr
	**Location**	Gunsan-si	Gunsan-si	Iksan-si	Iksan-si
	**GPS coordinates**	35°57′59.5″ N 126°46′56.1″ E	35°58′03.5″ N 126°46′51.5″ E	35°54′27.5″ N 126°58′04.1″ E	35°54′25.1″ N 126°58′03.2″ E
**Climatic conditions**	**MAT ^1^**	13.0 °C	13.0 °C	13.2 °C	13.2 °C
	**MAP ^2^**	1246 mm	1246 mm	1157 mm	1157 mm
**Soil characteristics**	**OM ^3^** ** [g/kg]**	84.22	59.65	35	33.62
	**Total N** ** [%]**	0.55	0.38	0.22	0.21
	**EC ^4^**	13.96	6.94	0.96	4.10
	**pH_CaCl2_**	5.2	7.1	8.0	7.5
	**K^+^** ** [cmol+/kg]**	5.55	2.89	1.35	1.46
	**Ca^2+^** ** [cmol+/kg]**	15.18	12.29	8.91	8.91
	**Mg^2+^** ** [cmol+/kg]**	5.45	6.11	2.73	3.48
	**Na^+^** ** [cmol+/kg]**	0.87	1.31	0.81	1.95
	**P_2_O_5_** ** [mg/kg]**	745.07	386.92	553.63	556.01
	**Total C** ** [%]**	4.89	3.46	2.03	1.95

^1^ Mean annual temperature; ^2^ Mean annual precipitation; ^3^ Organic matter; ^4^ Electrical conductivity.

## Data Availability

Dataset generated during the current study are available from the corresponding author on request.
